# *PTENP1* is a ceRNA for *PTEN*: it’s CRISPR clear

**DOI:** 10.1186/s13045-020-00894-2

**Published:** 2020-06-09

**Authors:** Marianna Vitiello, Monica Evangelista, Yang Zhang, Leonardo Salmena, Pier Paolo Pandolfi, Laura Poliseno

**Affiliations:** 1 Oncogenomics Unit, CRL-ISPRO, Pisa, Italy; 2 Institute of Clinical Physiology, CNR, Pisa, Italy; 3 Cancer Research Institute, Beth Israel Deaconess Cancer Center, Harvard Medical School, Boston, MA USA; 4 Department of Pharmacology and Toxicology, University of Toronto, Toronto, ON Canada; 5 Princess Margaret Cancer Centre, University Health Network, Toronto, ON Canada; 6 MBC, Department of Molecular Biotechnology and Health Sciences, University of Torino, Torino, Italy; 7 DRI (Desert Research Institute), Renown Health, Nevada System of Higher Education, Las Vegas, NV USA

**Keywords:** *PTENP1*, *PTEN*, ceRNA, CRISPR, CasRx-mediated knock-down, Cas9-mediated knock-in

## Abstract

Here we apply state-of-the-art CRISPR technologies to study the impact that *PTENP1* pseudogene transcript has on the expression levels of its parental gene *PTEN*, and hence on the output of AKT signaling in cancer. Our data expand the repertoire of approaches that can be used to dissect competing endogenous RNA (ceRNA)-based interactions, while providing further experimental evidence in support of the very first one that we discovered.

## Main text

In our 2010 paper entitled “A coding independent function of gene and pseudogene mRNAs regulates tumor biology”, we provided the first evidence that RNA molecules, including non-coding RNAs (such as pseudogenes) and mRNAs, may be endowed with a biological function that specifically relies on their ability to compete for microRNA binding [1].

Our findings have contributed to an evolving microRNA-RNA interaction paradigm, where RNAs are not only “passive” targets of microRNAs, but also “active” regulators of microRNA availability, through a mechanism termed competing endogenous RNA (ceRNA) [2, 3]. Since our publication, a plethora of mRNAs and non-coding RNAs (lincRNAs, pseudogenes, circular RNAs) have been reported to function as ceRNAs in vitro and in animal models. Furthermore, ceRNA functions have been demonstrated to go beyond individual RNA-RNA interactions and extend into complex transcript interaction networks that can be severely dysregulated in cancer [4, 5].

Subsequent to our 2010 publication, many studies independently confirmed *PTENP1* pseudogene as a ceRNA for *PTEN* in prostate cancer, in other cancer types (e.g., bladder cancer, breast cancer, clear cell renal cell carcinoma, endometrial carcinoma, gastric cancer, head and neck squamous cell carcinoma, hepatocellular carcinoma), and in other physio-pathological conditions (see Supplementary references for a list). Nonetheless, a number of articles published in prestigious journals have repeatedly raised concerns about this functional interaction. Herein, we wish to address those concerns raised regarding the techniques we used to modulate *PTENP1* expression and show its impact on PTEN expression.

To rule out potential non-specific effects associated with (1) supra-physiological expression of a 3′UTR [6–9] and (2) congestion of RNA interference machinery caused by siRNA transfection [7], we have chosen to downregulate *PTENP1* expression at the transcriptional or post-transcriptional level, taking advantage of CRISPR technology.

**Fig. 1:**
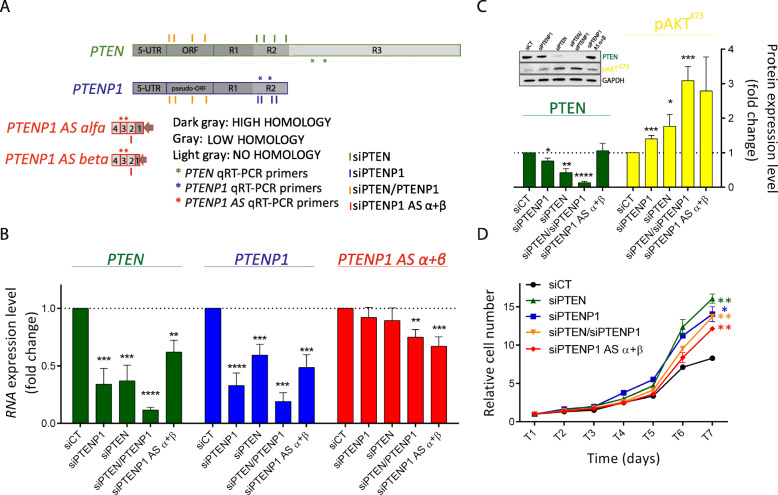
siRNA-mediated knock-down of *PTENP1* RNA results in the downregulation of PTEN expression and in an increase in DU145 cell proliferation. **a** Schematic representation of the location of the siRNAs used to downregulate *PTEN* (green), *PTENP1* (blue), *PTEN* + *PTENP1* (orange), and *PTENP1 AS α* + *β* (red) transcripts. The 5′UTR and open reading frame of *PTEN* and *PTENP1* are highly homologous (dark gray). Their 3′UTRs are composed of an R1 high homology region (dark gray), an R2 low homology region (gray), and an R3 region with no homology (light gray). The first exon of *PTENP1 AS α* and *β* is transcribed in antisense compared to the 5′UTR of *PTENP1*. The location of the siRNAs is chosen so that only the intended transcript(s) are selectively knocked down. The location of qRT-PCR primers used to detect *PTEN*, *PTENP1*, and *PTENP1 AS α* + *β* is indicated with green, blue, and red asterisks, respectively. **b** qRT-PCR quantification of *PTEN*, *PTENP1*, and *PTENP1 AS α* + *β* transcripts performed 24 h after the transfection of the indicated siRNAs. **c** (upper left) Representative western blot detection of PTEN, pAKT, and GAPDH proteins, performed 48 h after the transfection of the indicated siRNAs. (lower) Quantification of protein levels. **d** Growth curve of DU145 cells transfected with the indicated siRNAs. T1/2/3/4/5/6/7: days after the transfection. The results reported in **b**–**d** confirm the data reported in [1]: the knock-down of *PTENP1* negatively affects PTEN expression. Conversely, *PTEN* knock-down negatively affects *PTENP1* expression. As a consequence, AKT gets hyper-phosphorylated and cell proliferation increases. The graphs represent the mean ± SEM of three independent experiments. Statistically significant differences are indicated with asterisks: *p < 0.05, **p < 0.01, ***p < 0.001, ****p < 0.0001

To begin, we successfully replicated results reported in our original paper, in spite of the fact that the source of DU145 cells and the batch of siRNAs against *PTEN* and *PTENP1* were different, and that the experiments were performed in a different lab (Fig. 1).

**Fig. 2:**
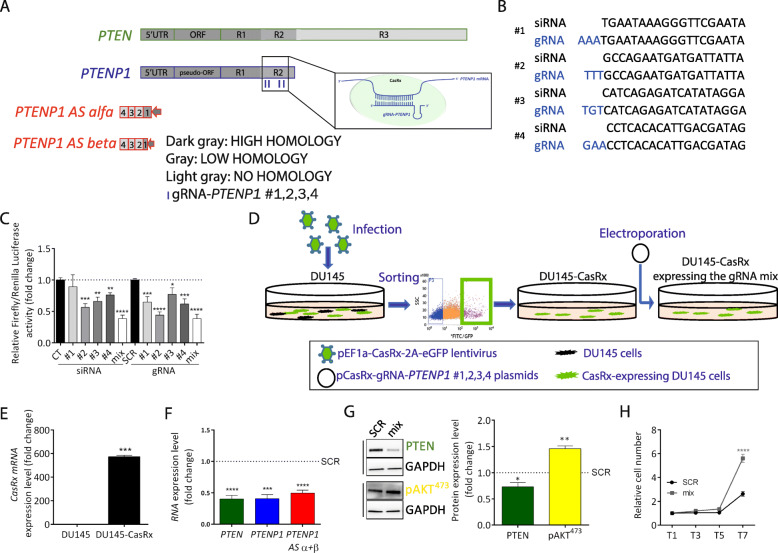
CRISPR-mediated knock-down of *PTENP1* RNA results in the downregulation of PTEN expression and in an increase in DU145 cell proliferation. **a**, **b** Schematic representation of the location of the 4 gRNAs used to knock down *PTENP1* expression (**a**). The sequence of *PTENP1* gRNAs (#1 to #4) corresponds to the sequence of the siRNAs described in Fig. 1, extended by 3 nt (blue, **b**). **c** Luciferase assay performed in HEK293T cells to compare the efficiency of single siRNAs and gRNAs, as well as their mixes, in downregulating the expression of *PTENP1*. The assay was performed 36 h after the co-transfection of pGLU/ψ3′UTR plasmid with the indicated siRNAs (siCT; siPTENP1 #1,2,3,4) or gRNA-expressing plasmids (pCasRx-gRNA (SCR); pCasRx-gRNA-*PTENP1* #1,2,3,4). In pGLU/ψ3′UTR plasmid, the R1 high homology and the R2 low homology regions of *PTENP1* 3′UTR are cloned downstream of Luciferase coding sequence [1]. **d** Cartoon summarizing the experimental protocol used to test the gRNAs in DU145 cells. DU145 cells were stably infected using the lentiviral vector pEF1a-CasRx-2A-eGFP, then sorted for high eGFP expression and, finally, electroporated with pCasRx-gRNA plasmid or the mix of pCasRx-gRNA-*PTENP1* #1,2,3,4 plasmids. **e** qRT-PCR quantification of *CasRx* mRNA in DU145 cells that were stably infected with pEF1a-CasRx-2A-eGFP and then sorted for high eGFP expression (DU145-CasRx). **f** qRT-PCR quantification of the indicated transcripts 24 h after the transfection of pCasRx-gRNA (SCR) or pCasRx-gRNA-*PTENP1* #1,2,3,4 mix in DU145-CasRx cells. **g** (left) Representative western blot detection of PTEN/GAPDH and pAKT/GAPDH proteins, performed 48 h after the transfection of pCasRx-gRNA (SCR) or pCasRx-gRNA-*PTENP1* #1,2,3,4 mix in DU145-CasRx cells. (right) Quantification of protein levels. **h** Growth curves of DU145-CasRx cells transfected with pCasRx-gRNA (SCR) or pCasRx-gRNA-*PTENP1* #1,2,3,4 mix. T1/3/5/7: days after the transfection. The results reported in **f**–**h** confirm the data obtained using siRNAs: the knock-down of *PTENP1* negatively affects PTEN expression. As a consequence, AKT gets hyper-phosphorylated and cell proliferation increases. The graphs represent the mean ± SEM of three independent experiments. Statistically significant differences are indicated with asterisks: *p < 0.05, **p < 0.01, ***p < 0.001, ****p < 0.0001

Next, in order to downregulate *PTENP1* post-transcriptionally, we used the recently reported CRISPR/CasRx system [10]. For this, we utilized 4 gRNAs designed on the same sequence of the 4 siRNAs composing the siPTENP1 mix (Fig. 2a, b) and we tested them for their ability to decrease the expression of a reporter construct in which *PTENP1* 3′UTR is cloned downstream of Luciferase coding sequence. As shown in Fig. 2c, the gRNAs work similarly to the corresponding siRNAs, with the mix of all 4 gRNAs working best. Therefore, we decided to use the combination of all 4, as we had done with siRNAs. In Fig. 2d–h, we show the results obtained upon the transient transfection of the gRNA mix in GFP-sorted DU145 prostate cancer cells that stably express CasRx-eGFP (Fig. 2d, e). Consistent with the RNA interference approach (Fig. 1), the gRNA mix caused a downregulation of the intended target *PTENP1* RNA, as well as of *PTEN* mRNA (Fig. 2f). The decrease in mRNA level was mirrored by a decrease in PTEN protein level and accompanied by increases in pAKT levels (Fig. 2g) and cell proliferation (Fig. 2h).

**Fig. 3:**
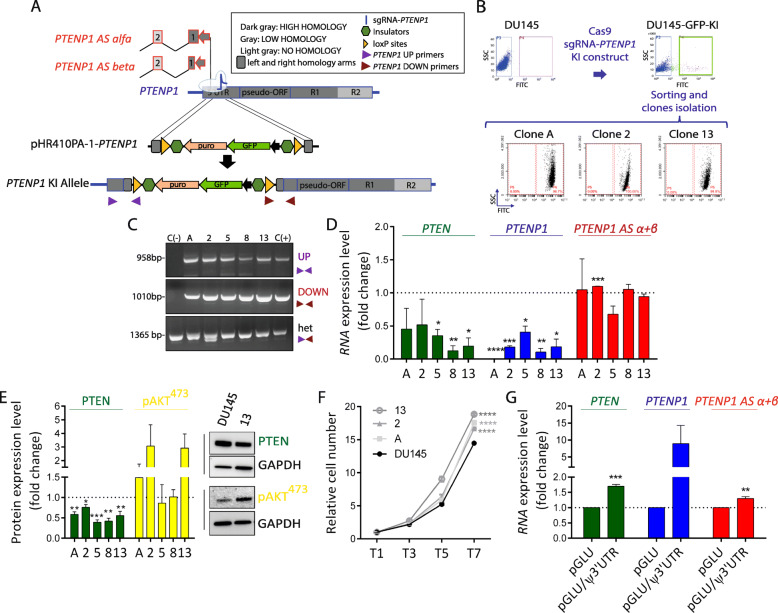
CRISPR-mediated knock-in of a GFP expression cassette in *PTENP1* gene results in the downregulation of PTEN expression and in an increase in DU145 cell proliferation. **a** Schematic representation of the CRISPR/Cas9-mediated cleavage of *PTENP1* gene region, followed by the homology recombination-mediated knock-in of a GFP expression cassette. Being oriented in the opposite direction, the cassette interferes with the transcription of *PTENP1* itself. The double-strand cut of genomic DNA produced by Cas9/sgRNA-*PTENP1* (blue) is located within the 5′UTR region of *PTENP1*, upstream of the promoter of *PTENP1 AS α* and *β*. Therefore, the transcriptional unit of the antisense transcripts is preserved. Besides the arms required for homology recombination and the GFP expression cassette, the knock-in construct contains 2 insulators and 2 loxP sites. **b** Cartoon summarizing the experimental protocol used. DU145 that constitutively express Cas9 and sgRNA-*PTENP1* were electroporated with pHR410PA-1-*PTENP1* plasmid that contains the knock-in construct. Then, Cas9-mediated cleavage of *PTENP1* genomic DNA was induced by adding doxycycline. After waiting 10–14 days in order to allow the dilution of non-integrated plasmid, cells that stably express GFP were sorted and seeded at 1-cell/96-well density to obtain individual knock-in clones. **c** PCR-based screening of the clones that are correctly knocked in. The junction regions located upstream and downstream of the knock-in construct were amplified using the primers shown in panel **a** as purple and dark red arrows, respectively. The purple forward and the dark red reverse primers (they give a PCR band only on wt alleles, not on knocked-in alleles) were also used to test whether the integration of the construct occurred on all chromosome 9 copies or not (het). The genomic DNA extracted from parental DU145 cells was used as negative control (C−). The genomic DNA extracted from DU145-GFP-KI cells right before 1-cell/96-well seeding was used as positive control (C+). Clones #A, 2, 5, 8, and 13 all show the correct integration of the construct, although non-knocked-in copies of chromosome 9 remain. **d** qRT-PCR quantification of the indicated transcripts in DU145 cells (taken as control) and clones #A, 2, 5, 8, and 13. **e** (left) Quantification of PTEN/GAPDH and pAKT/GAPDH protein levels in DU145 cells (taken as control) and in clones #A, 2, 5, 8, and 13. (right) Representative western blot detection of PTEN, pAKT and GAPDH proteins in DU145 cells and in clone #13. **f** Growth curves of DU145 cells and clones #A, 2, and 13. T1/3/5/7: days after seeding. **g** qRT-PCR quantification of the indicated transcripts 24 h after the electroporation of 1.5 μg of pGLU empty plasmid or of pGLU/ψ3′UTR plasmid in clone #13. The results reported in **d**–**g** confirm what obtained by RNA interference and CRISPR/CasRx: the knock-down of *PTENP1* negatively affects PTEN expression. As a consequence, AKT gets hyper-phosphorylated and cell proliferation increases. The graphs represent the mean ± SEM of three independent experiments. Statistically significant differences are indicated with asterisks: *p < 0.05, **p < 0.01, ***p < 0.001, ****p < 0.0001

We also adapted the CRISPR/Cas9-based gene replacement strategy [11] in order to achieve the downregulation of *PTENP1* at the transcriptional level. Specifically, we engineered an sgRNA-mediated cut between the promoter and the transcribed region of *PTENP1* gene. Then, by exploiting homology-mediated recombination, we “knocked-in” a GFP expression cassette in the reverse orientation, which interferes with *PTENP1* transcription (Fig. 3a). Using this strategy, we identified 11 GFP-positive KI clones (Fig. 3b), of which 7 harbored correct recombination of both homology arms and 5 showed the expected drop in *PTENP1* mRNA levels (clones #A, 2, 5, 8, and 13 reported in Fig. 3c, d). In these clones, we also observed a decrease in both PTEN mRNA and protein levels (Fig. 3d, e). In addition, clones #A, 2, and 13 had accompanying increases in pAKT levels (Fig. 3e) and cell proliferation (Fig. 3f). Crucially, in Fig. 3g, we show that endogenous *PTEN* mRNA levels are rescued in clone #13, if *PTENP1* 3′UTR is reintroduced by means of a plasmid that expresses it downstream of Luciferase coding sequence.

In summary, using 2 CRISPR-based technologies (Figs. 2 and 3), we confirmed our results achieved using RNA interference (ref. [1] and Fig. 1): knock-down of *PTENP1* leads to the repression of *PTEN* expression, hence the hyperactivation of oncogenic AKT signaling. In addition, we confirmed that siRNA-mediated knock-down of *PTENP1* antisense alpha + beta transcripts results in a downregulation of *PTENP1* and *PTEN* transcripts (Fig. 1b), as previously reported in [12]. Conversely, we showed that the knock-down of *PTEN* plus *PTENP1* transcripts by RNA interference (Fig. 1b) and of *PTENP1* by CRISPR/CasRx technology (Fig. 2f) represses the expression of *PTENP1* antisense transcripts, whereas the upregulation of *PTENP1* transcript elicits the opposite effect (Fig. 3g). In sum, we provide evidence that uncovers a dynamic cross-talk between *PTENP1* and *PTEN* sense transcripts on one side and antisense *PTENP1* transcripts on the other.

In the decade since our discovery, numerous groups have independently validated the regulatory interaction between *PTENP1* and *PTEN*. Altogether, these data provide a persuasive body of work to support the existence of a robust and reproducible functional interaction between this gene-pseudogene pair [13]. Finally, the new data presented herein further reinforces the *PTENP1-PTEN* paradigm and highlights the utility of CRISPR technologies for investigations of pseudogene-parental gene transcript relationships in cancer and other diseases.

## Supplementary information

**Additional file 1.** Supplementary references.

**Additional file 2.** Supplementary methods.

**Additional file 3.** Supplementary ** Figure 1. a** sgRNA-*PTENP1* sequence. **b** (left) *PTENP1* genomic sequence recognized by sgRNA-*PTENP1* (bold), and PAM sequence (5'-TGG-3', underlined). (right) Orthologous *PTEN* genomic sequence. sgRNA-*PTENP1* cannot mediate the cleavage of *PTEN* because of 4 mismatches (red), one of which falls in the PAM sequence. **c** Electropherogram of *PTENP1* genomic sequence, where the consequences of the cut by Cas9/sgRNA-*PTENP1* are shown. The electropherogram was obtained by PCR analysis of the genomic DNA extracted from DU145-Cas9/sgRNA-*PTENP1* double infected cells, 3 days after Cas9 induction using 2ug/ml doxycycline. The primers used for amplification were: Fw- attcgtcttctccccattcc; Rv-tctgcaggaaatcccatagc.

## Data Availability

Not applicable

## Abbreviations

ceRNA: competing endogenous RNA
CRISPR: Clustered regularly interspaced short palindromic repeats
