# Supplementary material for: PTENP1 is a ceRNA for PTEN: it’s CRISPR clear
Source: J Hematol Oncol. 2020 Jun 9;13:73. doi: 10.1186/s13045-020-00894-2 (PMC7285706; doi:10.1186/s13045-020-00894-2)
Supplement: Supplementary file 1 — Additional file 1. Supplementary references. [file 13045_2020_894_MOESM1_ESM.pdf]

SUPPLEMENTARY REFERENCES

Contexts in which *PTENP1* has been shown to act as sponge for *PTEN*:

-bladder cancer [1]

-breast cancer [2] [3] [4]

-clear cell renal cell carcinoma [5]

-endometrial carcinoma [6]

-gastric cancer [7]

-head and neck squamous cell carcinoma [8] [9]

-hepatocellular carcinoma [10] [11]

-prostate cancer [12] [13]

-other patho-physiological conditions [14] [15] [16] [17].

1. Zheng R, Du M, Wang X, Xu W, Liang J, Wang W, Lv Q, Qin C, Chu H, Wang M, et al: **Exosome-transmitted long non-coding RNA PTENP1 suppresses bladder cancer progression.** *Mol Cancer* 2018, **17:**143.
2. Li RK, Gao J, Guo LH, Huang GQ, Luo WH: **PTENP1 acts as a ceRNA to regulate PTEN by sponging miR-19b and explores the biological role of PTENP1 in breast cancer.** *Cancer Gene Ther* 2017, **24:**309-315.
3. Shi X, Tang X, Su L: **Over-expression of long non-coding RNA PTENP1 inhibits cell proliferation and migration via suppression of miR-19b in breast cancer cells.** *Oncol Res* 2017.
4. Gao X, Qin T, Mao J, Zhang J, Fan S, Lu Y, Sun Z, Zhang Q, Song B, Li L: **PTENP1/miR-20a/PTEN axis contributes to breast cancer progression by regulating PTEN via PI3K/AKT pathway.** *J Exp Clin Cancer Res* 2019, **38:**256.
5. Yu G, Yao W, Gumireddy K, Li A, Wang J, Xiao W, Chen K, Xiao H, Li H, Tang K, et al: **Pseudogene PTENP1 functions as a competing endogenous RNA to suppress clear cell renal cell carcinoma progression.** *Mol Cancer Ther* 2014.
6. Chen R, Zhang M, Liu W, Chen H, Cai T, Xiong H, Sheng X, Liu S, Peng J, Wang F, et al: **Estrogen affects the negative feedback loop of PTENP1-miR200c to inhibit PTEN expression in the development of endometrioid endometrial carcinoma.** *Cell Death Dis* 2018, **10:**4.
7. Zhang R, Guo Y, Ma Z, Ma G, Xue Q, Li F, Liu L: **Long non-coding RNA PTENP1 functions as a ceRNA to modulate PTEN level by decoying miR-106b and miR-93 in gastric cancer.** *Oncotarget* 2017, **8:**26079-26089.
8. Gao L, Ren W, Zhang L, Li S, Kong X, Zhang H, Dong J, Cai G, Jin C, Zheng D, Zhi K: **PTENp1, a natural sponge of miR-21, mediates PTEN expression to inhibit the proliferation of oral squamous cell carcinoma.** *Mol Carcinog* 2017, **56:**1322-1334.
9. Liu J, Xing Y, Xu L, Chen W, Cao W, Zhang C: **Decreased expression of pseudogene PTENP1 promotes malignant behaviours and is associated with the poor survival of patients with HNSCC.** *Sci Rep* 2017, **7:**41179.
10. Chen CL, Tseng YW, Wu JC, Chen GY, Lin KC, Hwang SM, Hu YC: **Suppression of hepatocellular carcinoma by baculovirus-mediated expression of long non-coding RNA PTENP1 and MicroRNA regulation.** *Biomaterials* 2015, **44:**71-81.
11. Qian YY, Li K, Liu QY, Liu ZS: **Long non-coding RNA PTENP1 interacts with miR-193a-3p to suppress cell migration and invasion through the PTEN pathway in hepatocellular carcinoma.** *Oncotarget* 2017, **8:**107859-107869.

12. Poliseno L, Salmena L, Zhang J, Carver B, Haveman WJ, Pandolfi PP: **A coding-independent function of gene and pseudogene mRNAs regulates tumour biology.** *Nature* 2010, **465:**1033-1038.
13. Johnson TS, Li S, Franz E, Huang Z, Dan Li S, Campbell MJ, Huang K, Zhang Y: **PseudoFuN: Deriving functional potentials of pseudogenes from integrative relationships with genes and microRNAs across 32 cancers.** *Gigascience* 2019, **8**.
14. Johnsson P, Ackley A, Vidarsdottir L, Lui WO, Corcoran M, Grander D, Morris KV: **A pseudogene long-noncoding-RNA network regulates PTEN transcription and translation in human cells.** *Nat Struct Mol Biol* 2013, **20:**440-446.
15. Wang L, Zhang N, Wang Z, Ai DM, Cao ZY, Pan HP: **Pseudogene PTENP1 Functions as a Competing Endogenous RNA (ceRNA) to Regulate PTEN Expression by Sponging miR-499-5p.** *Biochemistry (Mosc)* 2016, **81:**739-747.
16. Lai Y, Li J, Zhong L, He X, Si X, Sun Y, Chen Y, Zhong J, Hu Y, Li B, et al: **The pseudogene PTENP1 regulates smooth muscle cells as a competing endogenous RNA.** *Clin Sci (Lond)* 2019, **133:**1439-1455.
17. Wang Z, Song Y, Han X, Qu P, Wang W: **Long noncoding RNA PTENP1 affects the recovery of spinal cord injury by regulating the expression of miR-19b and miR-21.** *J Cell Physiol* 2019.
