# Supplementary material for: PTENP1 is a ceRNA for PTEN: it’s CRISPR clear
Source: J Hematol Oncol. 2020 Jun 9;13:73. doi: 10.1186/s13045-020-00894-2 (PMC7285706; doi:10.1186/s13045-020-00894-2)
Supplement: Supplementary file 2 — Additional file 2. Supplementary methods. [file 13045_2020_894_MOESM2_ESM.pdf]

SUPPLEMENTARY METHODS

Cells and culture. HEK293T cells were grown in DMEM low glucose medium plus 10% foetal bovine serum. DU145 were grown in RPMI medium plus 10% foetal bovine serum. Both cell lines were grown in 1% glutamine (Sigma-Aldrich) and 1% penicillin/streptomycin (Euroclone) containing medium, at 37°C in a humidified atmosphere with 5% CO_2.

Oligos. PCR and qRT-PCR oligos were purchased from Eurofins Genomics. The non-targeting siCT and the siRNAs for *PTEN* and *PTENP1* knock-down were purchased from Dharmacon. The siRNA for *PTENP1 AS α+β* knock-down was purchased from Eurofins Genomics. For sequences, see below:

Sequence of qRT-PCR primers

Gene	Forward primer	Reverse primer	ref
PTEN	GTTTACCGGCAGCATCAAAT	CCCCCACTTTAGTGCACAGT	PMID: 20577206
PTENP1	TCAGAACATGGCATACACCAA	TGATGACGTCCGATTTTTCA	PMID: 20577206
PTENP1 AS α+β	AAGGCTGCACTGTCAACTTCCCTA	TCCCACAATAGGTCTTCTGCAAGC	PMID: 23435381
CasRx	AGCTGACCAACTCCTTCTCC	AGCATCACTTCCCTGAGCTT	
GAPDH	CGCTCTCTGCTCCTCCTGTT	CCATGGTGTCTGAGCGATGT	PMID:28445987
PBGD	TCCAAGCGGAGCCATGTCTG	AGAATCTTGTCCCCTGTGGTGGA	PMID:28445987
SDHA	CCACTCGCTATTGCACACC	CACTCCCCATTCTCCATCA	PMID:28445987

Sequence of siRNAs

Gene	Sequence
siCT	#D 001210-02-20
siPTENP1 #1 *	TGAATAAAGGGTTCGAATA
siPTENP1 #2 *	GCCAGAATGATGATTATTA
siPTENP1 #3 *	CATCAGAGATCATATAGGA
siPTENP1 #4 *	CCTCACACATTGACGATAG
siPTEN #1 *	GGAAATTAGAGTTGCAGTA
siPTEN #2 *	ACTTATTGGTGCTGAAATT
siPTEN #3 *	GGCAAATAGATTACCCAGA
siPTEN #4 *	GATTCTACAGTAAGCGTTT
siPTEN/PTENP1 #1*	GAUCAGCAUACACAAAUUA
siPTEN/PTENP1 #2*	GACUUAGACUUGACCUAUA
siPTEN/PTENP1 #3*	GAUCUUGACCAAUGGCUAA
siPTEN/PTENP1 #4*	CGAUAGCAUUUGCAGUAUA
siPTENP1 AS α+β**	UGUACUGUCUGAUAUCUCC

* siRNA reported in PMID:20577206

** PTENpg1 asRNA Ex2 shRNA sequence reported in PMID:23435381

Plasmids.

<u>pRL-TK</u>. The pRL-TK plasmid expresses Renilla Luciferase and is cotransfected with pGLU plasmids as normalization control.

<u>pGLU/ψ3'UTR</u>. This plasmid expresses the R1 high homology and the R2 low homology regions of *PTENP1* 3'UTR downstream of Firefly Luciferase coding sequence. The empty pGLU plasmid is used as negative control [1].

<u>pXR001:pEF1a-CasRx-2A-eGFP</u>. This lentiviral vector, which expresses CasRx and eGFP from a bicistronic transcript, was purchased from Addgene (#109049).

<u>pCasRx-gRNA-*PTENP1* #1,2,3,4</u>. The 4 gRNAs against *PTENP1* (see **Fig.2b** for sequences) were cloned into pXR003:pCasRx-gRNA plasmid (Addgene, #109053), using BbSI restriction site. pCasRx-gRNA plasmid, which expresses a non-targeting gRNA, was used as control (SCR).

pCW-Cas9. This lentiviral vector, which expresses a doxycycline-inducible version of Cas9 and carries puromycin resistance, was purchased from Addgene (#50661).

pLX-sgRNA-*PTENP1*. In order to obtain pLX-sgRNA-*PTENP1* lentiviral vector, first of all the sequence of the sgRNA for *PTENP1* was chosen using CHOPCHOP online tool (https://chopchop.cbu.uib.no/) and double-checked for its inability to target *PTEN* gene **(Supplementary Fig.1a and b)**. Then, according to the protocol available at www.addgene.org, the PCR product corresponding to sgRNA-*PTENP1* was generated using as template pLX-AAVS1-sgRNA lentiviral vector (Addgene, #50662), Phusion Flash High-Fidelity PCR Master Mix (Thermo Scientific) and the following primers: F2 5'-G**CGGCAGCAGCTGCTGGATGG**GTTTTAGAGCTAGAAATAGCAA-3', R2 5'-**CCATCCAGCAGCTGCTGCCG**CGG-3'. Finally, the PCR product was cloned into pLX-AAVS1-sgRNA lentiviral vector itself, using XhoI and NheI restriction sites, so that pLX-sgRNA-*PTENP1* lentiviral vector was obtained. Being derived from pLX-AAVS1-sgRNA, this vector carries blasticidine resistance. The electropherogram reported in **Supplementary Fig.1c** confirms the ability of pLX-sgRNA-*PTENP1* to cut the intended region within *PTENP1* gene.

pHR410PA-1-*PTENP1*. This plasmid was used as template for homologous recombination, after Cas9/sgRNA-*PTENP1*-mediated cleavage at PAM site. The genomic regions located upstream and downstream of the PAM were amplified using the following primers: *PTENP1* upstream Fw 5'-catgtcgacctgaagcccctctcagcaacccg-3'; *PTENP1* upstream Rv 5'-atgggatccctactgggcctgcttctcctcagc-3'; *PTENP1* downstream Fw 5'-catagatctgcagctgctgccgcagccattacc-3'; *PTENP1* upstream Rv 5'- atggaattctatgcataaatcattataccag-3'. The amplified regions were then cloned into pHR410PA-1 plasmid (kind gift from Dr. Landi, University of Pisa, Italy) using SalI/BamHI (*PTENP1* upstream region) and EcoRI/BglII (*PTENP1* downstream region) restriction sites. In between the upstream and downstream homology regions, pHR410PA-1-*PTENP1* plasmid contains a GFP expression cassette flanked by 2 insulators and 2 loxP sites.

pMD2.G and pSpax2. These two plasmids were used for lentiviral packaging and they were cotransfected with pCasRx, pCW-Cas9 or pLX-sgRNA-*PTENP1* for the production of specific lentiviruses. They were a kind gift from Dr. Hernando, New York University, New York, USA.

siRNA transfection. DU145 cells (3.3×10^5) were seeded in 6-well dishes. The following day they were transfected with 100nM siRNAs using Dharmafect I according to the manufacturer's protocol. Six hours later, cells were trypsinized and seeded for the specific assays.

Plasmid electroporation. For plasmid electroporation, DU145 cells (1.5×10^6) were seeded in 10cm dishes. The following day, 10^6 cells were electroporated with 2ug of plasmidic DNA in total, using Amaxa Nucleofector™ II (Lonza) and following the manufacturer's protocol.

Viral infection. HEK293T cells were plated in 10cm dishes (3×10^6/dish) and 24h later they were transfected with 12ug of pEF1a-CasRx-2A-eGFP, pCW-Cas9 or pLX-sgRNA-*PTENP1*, 4ug of pMD2.G and 8ug of pSpax2 using PEI. Six hours later, the medium was changed. Virus containing media was collected at 48h and 72h after transfection. Then, it was filtered and the virus was pelleted using LentiX Concentrator (Takara, #631231). Once quantified, the virus was added to DU145 cells using 4ug/ml polybrene.

Generation of GFP knock-in clones. DU145 cells were stably infected with pCW-Cas9 and pLX-sgRNA-*PTENP1*. Once DU145-Cas9/sgRNA-*PTENP1* cells were obtained, they were electroporated with pHR410PA-1-*PTENP1* and then Cas9 was induced using 2ug/ml doxycycline (Merck, #D9891). 10-14 days later, green DU145-GFP-KI cells (i.e. cells in which the GFP cassette is stably inserted within the *PTENP1* gene) were sorted using FACSJazz (BD) and seeded in a 96-well plate at 1 cell/well density, in order to isolate individual KI clones.

Genomic DNA extraction and analysis of *PTENP1* genomic status. Genomic DNA was extracted from parental DU145 cells (used as negative control C(-)), unsorted DU145-GFP-KI cells (used as positive control C(+)) and KI clones (10^6 cells), following the protocol reported in [2]. *PTENP1* status was evaluated using two couples of primers: *PTENP1* UP primers that amplify the junction region located upstream of the knock-in construct (Fw: TCTCCTCTCAGAAGCTGCAG; Rv: GTGTCTGCAGGCTCAAAGAG (purple arrows in **Fig.3a**)) and *PTENP1* DOWN primers that amplify the junction region located downstream of the knock-in construct (Fw: GTGTCTGCAGGCTCAAAGAG; Rv: CGCCTCTGACTGGGAATAGT (dark red arrows in **Fig.3a**)). *PTENP1* UP Fw and *PTENP1* DOWN Rv were used to detect non knocked-in copies of chromosome 9.

Luciferase reporter assay. HEK293T cells were seeded at a density of 7.5×10^4 cells per 24-well dish. The following day, 50ng of pGLU/ψ3'UTR and 10ng of pRL-TK were co-transfected with 5nM of specific siRNAs or 300ng of pCasRx plus 500ng of pCasRx-gRNA-*PTENP1* #1,2,3,4 mix, using Lipofectamine 2000 (Life Technologies). Luciferase activity was measured 36 hours after transfection.

Real-time PCR (qRT-PCR). RNA was extracted using QIAzol reagent (Qiagen), following the manufacturer's instructions. RNA was subsequently quantified using Nanodrop Lite (Thermo Scientific). DNAse treatment and retrotranscription of the mRNA was performed using QuantiTect Reverse transcription Kit (Qiagen), according to the manufacturer's protocol and using a S1000 Thermal Cycler (Bio-Rad). qRT-PCR was performed with SsoAdvanced Universal Supermix (Bio-Rad) on a CFX96 Real-Time System (Bio-Rad).

Protein extraction and western blot. DU145 cells (10^6) were resuspended in 40ul of Lysis buffer (50mM Tris HCl, 1% TritonX100, 0.25% NaDeoxicholate, 1mM PMSF, 2mM Orthovanadate, proteinase inhibitors cocktail). Then proteins were extracted as described in [2]. The following antibodies were purchased from Cell Signaling and used according to the manufacturer's instructions: PTEN (#9559), pAKT-Ser^{473} (#4060) and GAPDH (#2118). Quantification of band intensities was performed using ImageJ software (http://rsb.info.nih.gov/ij/).

Growth curve. DU145 cells (7×10^3) were seeded in 12-well plates (3 wells per time point). Each time point was fixed with 4% PFA and stained using a crystal violet solution (0.1% crystal violet, 20% methanol, in water) as reported in [2]. Each sample was normalized on the time 1 (T1) sample and the data were graphed as cell percentage compared to time 1.

Statistical analyses. Data were analysed using unpaired t test (GraphPad Prism, GraphPad Software Inc.). The mean ± SEM of 3 independent experiments is reported. Values of $p<0.05$ were considered statistically significant (*$p<0.05$, **$p<0.01$, ***$p<0.001$, ****$p<0.0001$).

REFERENCES

1. Poliseno L, Salmena L, Zhang J, Carver B, Haveman WJ, Pandolfi PP: **A coding-independent function of gene and pseudogene mRNAs regulates tumour biology.** *Nature* 2010, **465:**1033-1038.
2. Vitiello M, Tuccoli A, D'Aurizio R, Sarti S, Giannecchini L, Lubrano S, Marranci A, Evangelista M, Peppicelli S, Ippolito C, et al: **Context-dependent miR-204 and miR-211 affect the biological properties of amelanotic and melanotic melanoma cells.** *Oncotarget* 2017, **8:**25395-25417.
