# Supplementary material for: PTENP1 is a ceRNA for PTEN: it’s CRISPR clear
Source: J Hematol Oncol. 2020 Jun 9;13:73. doi: 10.1186/s13045-020-00894-2 (PMC7285706; doi:10.1186/s13045-020-00894-2)
Supplement: Supplementary file 3 — Additional file 3. Supplementary Figure 1. a sgRNA-PTENP1 sequence. b (left) PTENP1 genomic sequence recognized by sgRNA-PTENP1 (bold), and PAM sequence (5'-TGG-3', underlined). (right) Orthologous PTEN genomic sequence. sgRNA-PTENP1 cannot mediate the cleavage of PTEN because of 4 mismatches (red), one of which falls in the PAM sequence. c Electropherogram of PTENP1 genomic sequence, where the consequences of the cut by Cas9/sgRNA-PTENP1 are shown. The electropherogram was obtained by PCR analysis of the genomic DNA extracted from DU145-Cas9/sgRNA-PTENP1 double infected cells, 3 days after Cas9 induction using 2ug/ml doxycycline. The primers used for amplification were: Fw- attcgtcttctccccattcc; Rv-tctgcaggaaatcccatagc. [file 13045_2020_894_MOESM3_ESM.pdf]

A

sgRNA-*PTENP1* CGGCAGCAGCTGCTGGATGG

B

PTENP1 gene

5’-CCA**CCATCCAGCAGCTGCTGCCG**-3’
3’-GGTGGTAGGTCGTCGACGACGGC-5’

PTEN gene

5’-CAA**CCATCCAGCAGCCGCCGCAG**-3’
3’-GTTGGTAGGTCGTCGGCGGCGTC-5’

C

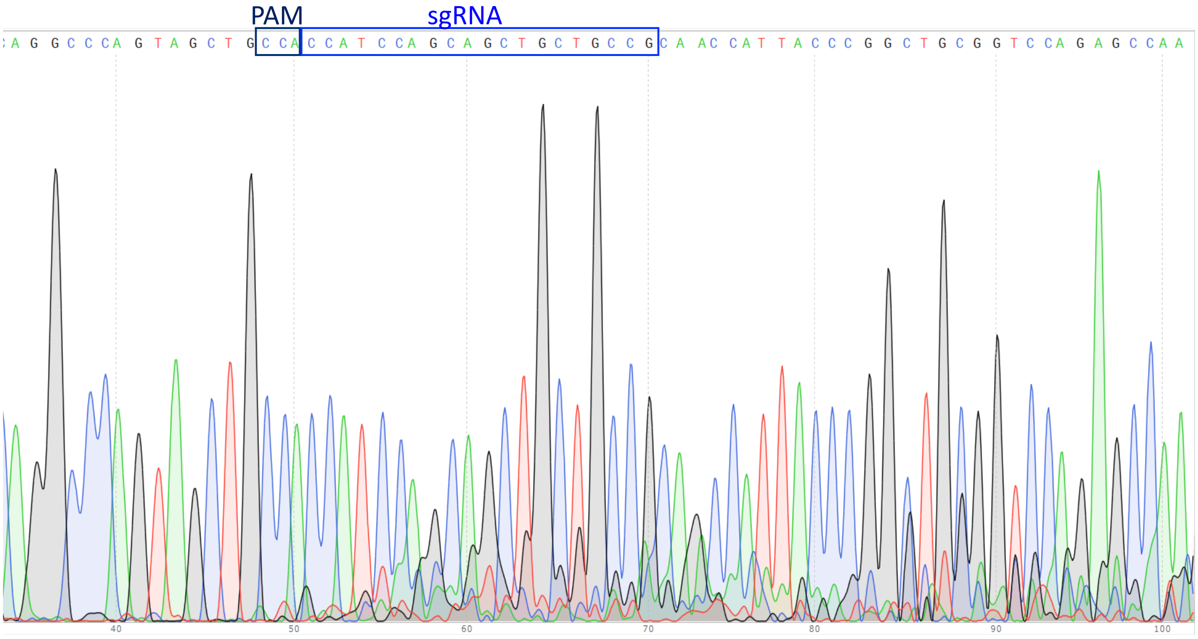

Supplementary Figure 1.

a sgRNA-*PTENP1* sequence. **b** (left) *PTENP1* genomic sequence recognized by sgRNA-*PTENP1* (bold), and PAM sequence (5’-TGG-3’, underlined). (right) Orthologous *PTEN* genomic sequence. sgRNA-*PTENP1* cannot mediate the cleavage of *PTEN* because of 4 mismatches (red), one of which falls in the PAM sequence. **c** Electropherogram of *PTENP1* genomic sequence, where the consequences of the cut by Cas9/sgRNA-*PTENP1* are shown. The electropherogram was obtained by PCR analysis of the genomic DNA extracted from DU145-Cas9/sgRNA-*PTENP1* double infected cells, 3 days after Cas9 induction using 2ug/ml doxycycline. The primers used for amplification were: Fw-attcgtcttctccccattcc; Rv-tctgcaggaaatcccatagc.
